# Physiological and Psychological Effects of Parent-Delivered Traditional Thai Massage in Children With Autism: Protocol for a Randomized Controlled Trial

**DOI:** 10.2196/41839

**Published:** 2023-02-08

**Authors:** Hui Ruan, Wichai Eungpinichpong, Hua Wu, Chanada Aonsri

**Affiliations:** 1 Graduate School Khon Kaen University Khon Kaen Thailand; 2 Research Center in Back, Neck, Other Joint Pain and Human Performance Division of Physical Therapy Khon Kaen University Khon Kaen Thailand; 3 School of Physical Education Hainan Normal University Haikou China; 4 Department of Special Education Khon Kaen University Demonstration School Khon Kaen University Khon Kaen Thailand

**Keywords:** autism, massage, randomized controlled trial, protocol

## Abstract

**Background:**

Although many autistic children receive massage as a complementary therapy, it is not included in evidence-based practice for autism because evidence of its efficacy is lacking. Further, prior studies have failed to identify objective indicators of core symptoms or elucidate their mechanisms.

**Objective:**

We developed a parent-delivered traditional Thai massage (TTM) intervention for children with autism, aiming to experimentally determine whether children with autism truly experience positive effects from parent-delivered TTM and determine possible mechanisms of the observed effects.

**Methods:**

A 2-armed, parallel randomized controlled trial was conducted between February 2022 and June 2022. Forty-eight children with autism (aged 7-12 years) were recruited from the Hainan Special Education School and randomly assigned to either a parental TTM or control group at a ratio of 1:1 based on random numbers generated with Online Research Randomizer. The generated sequences were concealed in an opaque envelope. Individuals in the parental TTM group received 16 parent-delivered TTM sessions over 8 weeks at the school’s health room after school, and the control group maintained a normal daily routine. Outcomes were assessed on admission, after 8 weeks, and at a 2-month follow-up and included the effect of massage treatment on autism symptoms, measured with the Autism Treatment Evaluation Checklist score (evaluated by parents and a blinded teacher), physiological parameters (ie, heart rate variability and gait), and the Parenting Stress Index, Fourth Edition–Short Form.

**Results:**

We finished all data collection on June 20, 2022. Data analysis will be started, and we expect to publish results in 2023.

**Conclusions:**

This study will provide further evidence for massage treatment of autism and provide support for family-based care.

**Trial Registration:**

Chinese Clinical Trial Registry ChiCTR2100051355; https://tinyurl.com/3dwjxsw5

**International Registered Report Identifier (IRRID):**

DERR1-10.2196/41839

## Introduction

### Background

Autism is a neurodevelopmental disorder that affects central nervous system development. The core symptoms are characterized by social interaction and social communication deficits and restrictive-repetitive behavior patterns [1]. The disorder is not associated with any particular culture, ethnicity, race, or socioeconomic group [2]. The majority of individuals with autism also have motor disorders, sensory disorders, anxiety, hyperactivity, sleep disorders, or autonomic nervous system (ANS)-associated disorders [3-5]. The prevalence of autism has risen rapidly in the last decade. An assessment commissioned by the World Health Organization in 2012 estimated the global prevalence of autism to be approximately 1% [6]. An updated report from the US Centers for Disease Control and Prevention showed that 1 in 44 children aged 8 years was estimated to have autism spectrum disorder (ASD) [7]. A survey found that the incidence of autism among children in China was comparable to that in Western countries [8].

A variety of motor disorders are common in autism [9]. Postural [10] and gait abnormalities [11,12] can appear as early as infancy [13] and persist into childhood [14,15] and adulthood [16]. Previous studies showed that motor impairment may result in atypical movement [17] and limit social interaction [18] due to sensory integration impairment [16]. This could, in turn, lead to the development of core autistic symptoms [18,19] and reduced quality of life [20].

The core symptoms of autism can be explained in part by ANS dysregulation [3,21,22]. Messina et al [23] found sympathetic activity, such as heart rate variability (HRV; the variation in time between each heartbeat), which is controlled by the ANS, may be considered an important tool for autism assessment. Measurements of HRV are considered to be the standard noninvasive method for assessing ANS function [23] and have also been suggested to be a valuable situation-specific marker of the effective regulation of children’s binary social interactions [24]. Previous reports showed that tonic HRV was significantly reduced in children with ASD compared with typically developing children [22,25]. Lory et al [25] suggested that ANS disorders in autism may lead to atypical attentional responses to sensory stimuli.

Atypical sensory responses (hyper- or hyporeactivity to sensory stimuli) are present in 90% of children with autism [26] and have been listed in the core diagnostic criteria for autism in the Diagnostic and Statistical Manual of Mental Disorders, 5th Edition, with abnormal tactile responses commonly reported [27]. Thye et al [19] reviewed the evidence of a relationship between sensory and social behavior in autism, especially in terms of touch and tactile processing irregularities, and concluded that it may be associated with social dysfunction.

### Prior Work

Massage is a touch-based therapy [28] defined as the use of the hands, feet, arms, or elbows to touch or manipulate the body surface of the person being massaged for healing and maintenance purposes [29]. Massage originates from human instinct and was developed as a kind of technology by the ancients. Historical records of massage therapy date back 3000 years, and massage therapy is mentioned in the earliest medical book in China, the *Huangdi Neijing* (*The Yellow Emperor’s Classic of Internal Medicine*), which recorded the theory of Chinese medicine and massage theory. The value of massage as a form of therapy has been recognized throughout history by ancient civilizations in China, India, Japan, Thailand, and, later, the ancient Egyptians, as well as the Greeks and Romans [30]. Since then, massage therapy has been continuously developed and applied, and is widely used in clinical practice to relieve pain, eliminate fatigue, and promote rehabilitation. A review by Field [31] outlined the clinical implementation of massage theory in recent years. Massage has recently been employed as a complementary therapy in the treatment of autism. It has been reported that 11% to 16% of people with autism receive massage therapy [32]. Initial studies in this area of research suggest that massage might have positive impacts on social communication [33,34], stereotypic behavior [35,36], sensory profiles [37,38], and language ability [39,40] in autism. According to research findings, positive touch stimuli can increase oxytocin release [41,42]. Oxytocin is known to be involved in ANS circuit regulation [41], which affects both emotional and social processes [43]. Suitable pressure stimulation is the critical feature of massage, and stimulation of pressure receptors has been shown to result in increased vagal activity. Increased vagal activity is associated with slower heart rate and enhanced attentiveness [31].

Traditional Thai massage (TTM) is believed to have ancient origins and has 3 distinctive features: deep pressure massage, work on meridian (energy) lines, and stretching of the affected muscles and joints. TTM is widely used in health care [44,45] and rehabilitation [46,47]. The results of studies conducted to date suggest that TTM may facilitate immediate and short-term improvement in HRV [48,49] and some gait parameters in nonautistic patients [50,51]. Since an increase in HRV reflects a state of relaxation due to parasympathetic activity induced by TTM, patients either with or without autism can relax and reduce their restrictive-repetitive behavior patterns. Consequently, they should improve their gait pattern and speed. TTM has also been effectively used in the rehabilitation of children with disabilities by the Bangkok Foundation for Children with Disabilities [52]. However, we only found one study [53] that reported the effects of TTM administered by masseurs on autistic behavior, and its methodology and conclusions were questionable [54]. It appears that more research is needed to explore the effects of TTM on autism by using both subjective and objective evaluation.

Furthermore, the refractory and lifelong nature of autism has profound implications for parents of autistic children and evidence shows that parents of children with autism experience more parenting stress than parents of nonautistic children or children diagnosed with other disabilities [55,56]. One recent review suggested that family-based interventions for mental disorders in children are becoming increasingly important, with more positive outcomes when parents are involved in the treatment [57]. Previous research revealed that parents who engaged in a massage-based intervention not only satisfied family-based care needs [58] but also showed relieved anxiety [42] and strengthened parent-child bonding [37,38,59]. The effects of TTM delivered by the parents of autistic children on these outcome measures have not been verified. This study aims to assess the effects of parental-delivered TTM in children with autism by evaluating changes in symptoms and physiological parameters (ie, gait and HRV). The primary objective of the study is to assess the effectiveness of parental TTM in improving symptoms of autism according to the Autism Treatment Evaluation Checklist (ATEC). Secondary objectives are to evaluate instant and cumulative effects of the parental TTM intervention via an assessment of gait and HRV and explore the relationship between symptom improvement and observed changes in physiological parameters. The tertiary objective is to examine changes in levels of parental stress after parents have administered the TTM intervention. The following four research questions will be addressed: (1) Do core symptoms significantly improve (ie, does ATEC score improve) in autistic children after parental TTM in comparison with the control group? (2) Do HRV and gait indicators significantly improve in children with autism after parental TTM? (3) Is ATEC score improvement related to observed changes in physiological parameters? (4) How does parental stress change throughout the intervention period, as measured by the Parenting Stress Index, Fourth Edition–Short Form (PSI-4-SF)?

## Methods

### Participants

#### Inclusion Criteria

The following inclusion criteria were applied to select autistic children: they had autism diagnosed by a child psychologist, were aged 7 to 12 years, were not already receiving systemic massage therapy, and were able to follow instructions. The children’s primary caregivers met the following criteria: they had been providing ≥1 year of childcare, were able to communicate with the massage trainer, and were able to read the questionnaire. Teachers met the following inclusion criteria: they served as primary caregivers for autistic children at school, had provided care for at least one semester, and were willing to participate in research.

#### Exclusion Criteria

This study applied exclusion criteria to 3 groups of people: the autistic children, their parents, and their teachers. For autistic children, the following exclusion criteria were applied: contraindications to TTM, such as fractures, fever, or psychoactive medication, and administration of other specialized interventions during the experimental period. Parents were excluded if they were not available at the allotted time. Further, teachers with insufficient time to complete the questionnaire were excluded.

#### Sample Size

The sample size calculation was based on research by Najafabadi et al [60]; no previous study has used both an autism massage intervention and the ATEC scale. The standard deviation of the social ATEC scores of the 2 groups was used to calculate the sample size needed to achieve a power of 80% and a 2-sided significance level of 5%. A dropout rate of 10% was assumed to determine the principal of intention for estimating the final sample size needed. According to a previously reported formula [61], a total of 48 participants were recruited for the study.

### Design and Setting

**Figure 1:**
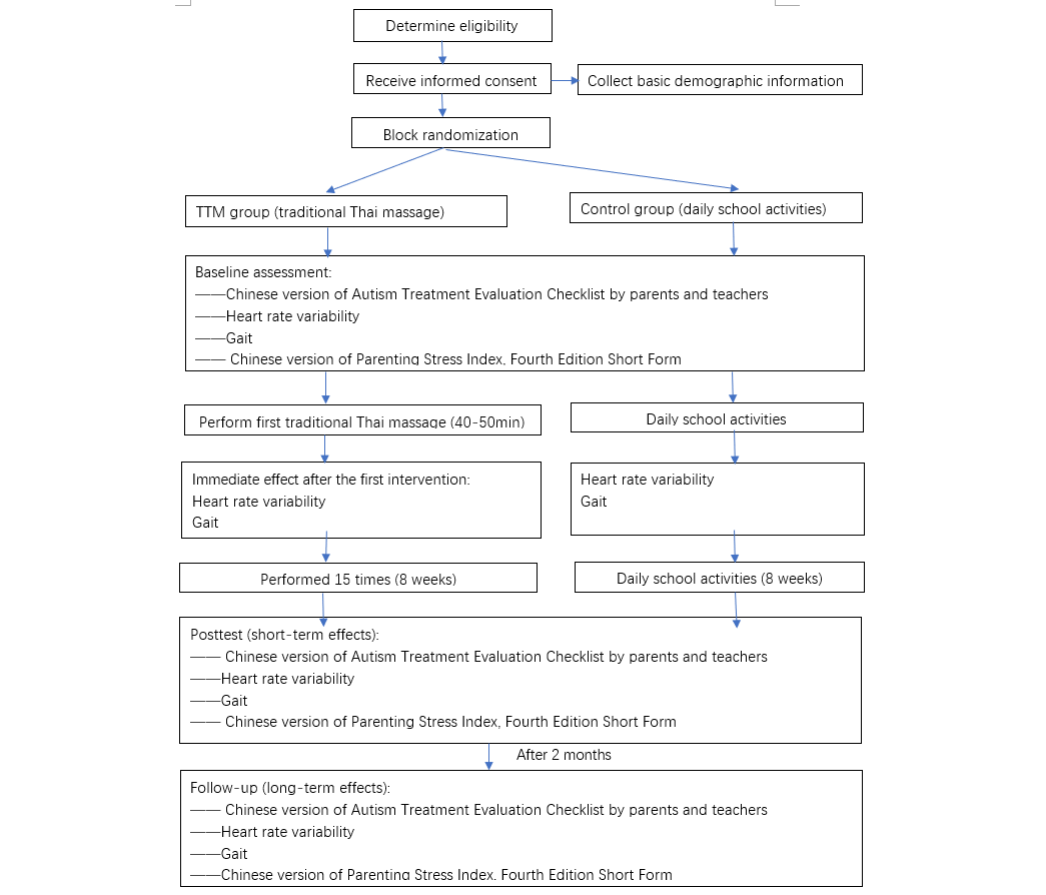
Flowchart depicting the study design.

A randomized controlled trial (RCT) was conducted between February 2022 and June 2022. Participants were recruited from the Hainan Special Education School in Hainan Province, China. A recruiting poster and presentation were posted on the bulletin board at the school. Figure 1 depicts a flowchart of the study protocol prepared according to the SPIRIT (Standard Protocol Items: Recommendations for Interventional Trials) guidelines [62] (Multimedia Appendix 1). Before recruiting the participants, informed consent forms (Multimedia Appendix 2) were sent to the parents via the teachers, and if the parents agreed to participate, they signed the form and submitted it to a designated box within 2 days. The children with autism who could communicate with their parents also gave either verbal or written informed consent.

#### Randomization

Forty-eight participants (aged 7-12 years) from grades 1 to 6 were randomly assigned to parental TTM and control groups by random numbers generated with Online Research Randomizer (Geoffrey C Urbaniak and Scott Plous). The random numbers were then concealed in an opaque envelope.

#### Blinding

Since the massage intervention took place after school hours, parents brought their children to the health room, which is located far from the teaching building. Teachers were blinded to the group assignments of the children included in the study. Parents were asked to refrain from discussing the intervention to prevent contamination of the control group. Data collectors were also unaware of group placement.

### Ethical Considerations

This study complies with the ethical standards of the relevant national and institutional guidelines on human experimentation and was approved by the Khon Kaen University Ethics Committee for Human Research (HE642167). The study followed the Helsinki Declaration. Results will be published in a peer-reviewed journal and presented at domestic and international academic conferences to further promote communication. This protocol was registered with the Chinese Clinical Trial Registry (ChiCTR2100051355).

Researchers provided parents with information about the study and obtained informed consent from them while their children were in school. The parent or guardian signed the document and returned it to the school researcher or the teacher. Before the study, the parents were responsible for asking their children about their willingness to participate in the study. Children whose parents provided informed consent took part in the study. The researchers explained the study methods verbally to the children and parents and demonstrated the tests that would be performed on the children; the teachers and parents observed each child’s reaction to determine if the child consented or not before the test.

In the massage group, the participants were paid US $7 for each TTM session. In the control group, each volunteer participated in the evaluation 4 times and was paid US $7 each time. The decision was made that after data collection was finished, if good results were obtained in the parental TTM group, the control group would also be given training in TTM. Each teacher volunteer who participated in the 3 stages of assessment received US $30 for each stage.

### Intervention

Children assigned to the TTM group received sixteen 40-to-50-minute parent-delivered TTM sessions over an 8-week period (2 sessions per week) in the health room of the school after school had finished. Gifts and rewards were given to parents and children to increase intervention adherence rates. The control group maintained a normal daily routine when in school.

#### TTM Training

The parents of autistic children who were enrolled in the study took part in 3 TTM training sessions. Each 3-hour session was hosted by experts at the health room of Hainan Special Education School. Parents of participants in the intervention group were trained in TTM by an experienced traditional Thai masseur with a massage qualification until they became able to perform the experimental intervention. Thereafter, the massage trainer monitored the parents to ensure that they continued to perform TTM properly at each session.

#### TTM Procedure

The TTM method involves pressure massage and muscle stretching. Pressure massage involves massaging with the thumbs and pressing with the palms until slight muscle resistance is felt. The children may feel slight muscle tightness, but the massage should not be painful. The masseur will maintain pressure for 5 to 10 seconds and then release it to allow energy or blood to flow freely around the massage area; this is repeated 2 to 3 times for each body part. Muscle stretching includes the masseur observing the children and asking them whether they feel that the stretch is too deep; if the stretch is acceptable, they hold the appropriate position for 15 to 30 seconds [63]. The 9 steps of TTM are based on standard practice. However, they have been modified for this patient population: gentle touch is used to facilitate relaxation and promote social communication, motor control, and a sense of confidence in and love for the children. Starting from the head and continuing to the limbs easily promotes relaxation. Stretching at the end of the session is one of the principles of TTM.

**Figure 2:**
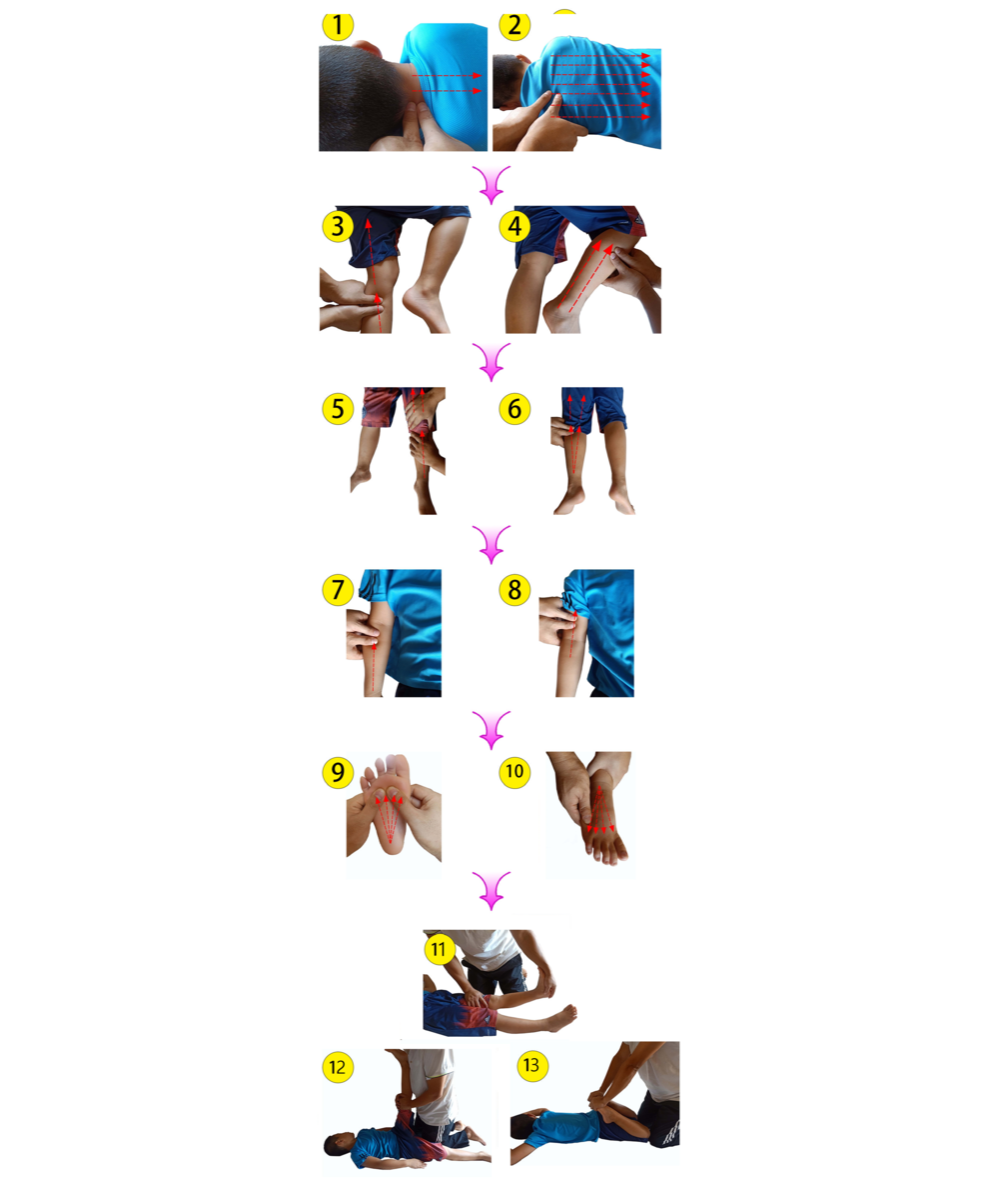
Traditional Thai massage procedure: (1) neck massage, (2) back and waist massage, (3) inner thigh massage, (4) outer leg massage, (5) leg massage in the supine position, (6) leg massage in the prone position, (7, 8) arm massage, (9, 10) foot massage, and (11-13) muscle stretching. The procedure is performed in the numbered order.

In the starting position, the children lie on their side with the upper leg bent at the knee forming a 90° angle with a pillow placed under the head and a straight lower leg. The parent performing the massage kneels or sits behind the child. Massage strategies for distinct massage types should be performed as described in Table 1 and Figure 2.

**Table 1:** Traditional Thai massage procedures for children with autism and the order in which they are performed.

Target area	Description of procedure
1. Neck (lateral position)	Massage is started in the neck region and extends along the back muscles toward the lumbar area. Massage is performed along the side of the neck toward the shoulders.
2. Back and waist	The paravertebral muscles, which are close to the thoracolumbar spine, are massaged toward the waist level. There are 3 lines of massage. One is started on the upper back near the spinous processes of T1 to L5 on each side of the back. The second line is about 1 cm along the side of the first one. The third line is about 1 cm along the side of the second one. A pressing massage style should be applied on the 3 lines, which should be initiated in the thoracolumbar region and continue toward the top of the sacrum.
3. Inner thigh (straightened legs)	Thumb pressure should be applied to the medial side of a straightened leg from the Achilles tendon toward the groin (along the line in panel 3 of Figure 1). This type of massage should be performed along 2 massage lines. The first massage line extends along the medial side of the leg, and the second massage line extends along the backside of the leg.
4. Outer leg massage (on the bent leg side):	Both hands are placed on the lower leg of the child. The lateral part of the lower leg should be pushed down using the thumb. This massage style should be applied from the area above the lateral malleolus and continued to the side of the knee joint. The top of both knees are pressed toward the middle of the hip. Outer leg massage should be performed along the 2 massage lines. The first line extends along the front of the leg, and the second line is positioned slightly behind the first line.
Repeat on opposite side	The child lies on the other side and procedures 1 to 4 are repeated.
5. Leg (supine position)	Massage is performed in front of the ankle to near the groin.
6. Leg massage (prone position)	Massage is initiated behind the heel around the Achilles tendon and continues toward the gluteal and hamstring muscles.
7. Arm	With the child in a prone position, thumb pressure is applied along the back of the lower and upper arm toward the shoulder. The procedure is then repeated on the other arm. In the supine position, push massage is applied upward from the center of the forearm and along the center of the arm toward the axilla.
8. Foot (supine position)	A foot massage is performed by applying pressure from the heel to all 5 toes. Then, the tip of each toe should be pulled gently.
9. Muscle stretch (the appropriate position is held for 15 to 30 seconds)	With the child in a supine position, the heel is stretched and the child’s foot is lifted until tension is felt. In the prone position, the child’s knees are flexed as much as possible by applying gentle pressure to the soles of the feet.

### Fidelity

The fidelity measurement represents the number of times parents use massage with their children, incorporating any adjustments to the massage technique made by the expert. More than 90% attendance is expected, and the expert will correct improper technique in real time.

### Primary Outcome Measurement: ATEC

The ATEC questionnaire was developed by Dr Bernard Rimland and Dr Stephen M Edelson at the Autism Research Institute in the United States in the mid-1990s to help researchers and parents evaluate the effectiveness of various treatments for autistic children and adults [64]. It is not used to diagnose autism. It comprises 4 subscales: speech, language, and communication; sociability; sensory and cognitive awareness; and health and physical behavior. The maximum score is 179, with higher scores indicating autism of increased severity. The sensitivity and specificity of the Chinese version of the ATEC total scale and its subscales have been determined to range from 0.922 to 0.987 and 0.803 to 0.887, respectively; the scale has also been determined to have high reliability [65].

### Secondary Outcome Assessments

#### Gait

Gait was measured using a wearable BTS G-Walk device (BTS Bioengineering Company). Experimental gait data were collected at a frequency of 100 Hz and transmitted to a computer via a Bluetooth connection. All gait parameter measurements have been determined to have high test-retest and intertrial reliability (intraclass correlation coefficient: 0.728-0.969 and 0.84-0.99, respectively) [66,67]. Wireless internet was required for the gait tests, which were carried out on a flat surface that measured at least 10 × 10 m. Prior to performing the test, the height of the participant was entered, and an adjustable elastic band was placed around the participant’s waist. When taking the test, participants stood in place for a few seconds. After confirming that the sensor was connected to the computer via Bluetooth and the connection was stable, each child volunteer followed a marked 7-m path back and forth while walking normally. Teachers or parents were allowed to provide verbal cues but not physical assistance.

#### HRV Measurement

UBiomacpa (version 1; Biosense Creative) was used to detect the activity of the autonomic nervous system. The HRV test was carried out in an independent, quiet room at an ambient temperature of 26 °C. Before starting the test, the tester ensured that the equipment was properly connected. After receiving TTM, the autistic children were placed in a sitting position with a pulse sensor attached to their left index finger, and they underwent an HRV test for 2 minutes that was administered by a physiotherapist. When the test was complete, the device automatically generated a data report via a connected computer.

#### PSI-4-SF Assessment

The PSI-4-SF consists of 36 items in the following 3 dimensions: parent distress, parent-child dysfunction interaction, and difficult child [68]. The Chinese version of the PSI-4-SF has an internal consistency and reliability that ranges between 0.92 and 0.95 for each subscale. For the whole scale, the value is 0.97, indicating that this scale has good consistency and stability [69].

### Data Collection, Management, and Analysis

IBM SPSS (version 25; IBM Corp) will be used for data analysis. Missing outcome data will not be imputed in the analysis, but the multiple imputation method will be used to explore the potential impact of missing data on outcomes. Demographic data will be expressed as means with standard deviation and percentages. *P*<.05 will be considered statistically significant. Holm correction of *P* values will be applied for multiple comparisons to compare the immediate effects of TTM for autistic children and effects in the short and long term. The primary outcome is the ATEC score, which will use generalized estimating equations. Time will be the covariate and the model will include group, time, and the group × time interaction. A 2-tailed paired *t* test or Wilcoxon signed-rank test will be used to test ATEC score, HRV, and gait parameters, as well as PSI-4-SF score changes within each group after testing for normal distribution. The relationship between physiological effects and ATEC results will be evaluated using a correlation test. A coefficient value <0.4 indicates a low degree of correlation, a value between 0.4 and 0.7 indicates a medium degree of correlation, and a value >0.7 indicates a high degree of correlation between variables.

Two trained assessors will be responsible for data collection, electronic double entry of the data, and management of the database. A team from the Key Laboratory of Data Science and Smart Education at Hainan Normal University, which is independent of the study team, will be responsible for data monitoring.

### Harms

No adverse events from TTM interventions have been reported in previous studies. Furthermore, in both intervention and control groups, data collection did not cause physical harm to the children and did not create psychological, social, or financial risks. If there is a risk of affecting the body (ie, pain or injury) or mind (ie, dissatisfaction, discomfort, hate, or boredom) of any participant, we decided to temporarily suspend or stop that individual’s participation and data gathering. Moreover, if any severe adverse events, such as death or other medical occurrences resulting in hospitalization, were to occur during the massage intervention sessions, the researcher planned to terminate the intervention and immediately report the event to the nearest local hospital in China. The researchers will report whether any such adverse events occurred to the ethics committee as required and describe them in the final paper.

### Confidentiality

The research group will keep the data confidential, and the research group will use passwords to record and access data. In this study, only the research group will have access to the data, and the research group will not state the names of the participants under any circumstances if the study results are published in a journal. The participants’ personal information will be stored in a specific file with a preset code.

### Ancillary and Posttrial Care

Participants could contact the researchers during the trial or 1 month after participation ended. Plans were made for participants with serious adverse events to receive a timely and appropriate response.

### Patient and Public Involvement

The patients and public will not be involved in the design, conduct, reporting, or dissemination of this research.

## Results

We finished all data collection on June 20, 2022. Data analysis will be started, and we expect results to be published in 2023. The normal distribution of the data will be determined before descriptive statistics for all variables are calculated. The participants’ demographic information will be reported as descriptive data. The primary outcomes will be expressed as continuous variables, and generalized estimating equations will be used to evaluate the effect of TTM on the children (via the ATEC score) at 3 time points: immediate, short term, and long term. Changes in physiological parameters (ie, HRV and gait parameters) after the intervention will be analyzed. Further, the relationship between ATEC score and physiological parameters will be identified with a correlation analysis. Finally, changes in parental stress throughout the intervention period will be investigated with the PSI-4-SF.

## Discussion

This study aims to assess the effects of parent-delivered TTM in children with autism by evaluating changes in symptoms and physiological parameters (ie, gait and HRV). In practice, massage has previously been used to treat autism. However, this treatment has not been included in evidence-based practice for autism due to the lack of long-term, extensive scientific research to supports its use. At present, studies of interventions have mainly focused on qigong massage and tui na massage. Furthermore, we found that outcome measures in most prior studies were based on questionnaires and scales evaluated before and after the intervention, and that there was a lack of studies that explored the mechanisms that underlie core symptom improvement. Presumably, massage adjusts the body and mind via sensory processes [70,71] and neural and hormonal regulation (eg, changing oxytocin levels).

In comparison with prior work, this will be the first RCT to explore parental TTM in terms of its physiological and psychological effects on children with autism. We will use objective indices to investigate possible mechanisms underlying the observed effects. Our study may highlight the value of complementary and alternative therapies for adjunctive treatment of autism to enhance family-based interventions. Both parents and blinded teachers will be evaluated for effects that might cause bias. Finally, the variability of massage manipulation by parents was minimized by expert training, examinations, and supervision.

## Appendix

Multimedia Appendix 1 SPIRIT guidelines.

Multimedia Appendix 2 Parents’ consent form.

## Abbreviations

ANS: autonomic nervous system
ASD: autism spectrum disorder
ATEC: Autism Treatment Evaluation Checklist
HRV: heart rate variability
PSI-4-SF: Parenting Stress Index, Fourth Edition–Short Form
RCT: randomized controlled trial
TTM: traditional Thai massage

## Data Availability

Data sharing is not applicable to this study as no new data were generated or analyzed. The data sets generated or analyzed after the experiments will be made available from the corresponding author on reasonable request.
