# Supplementary material for: Physiological and Psychological Effects of Parent-Delivered Traditional Thai Massage in Children With Autism: Protocol for a Randomized Controlled Trial
Source: JMIR Res Protoc. 2023 Feb 8;12:e41839. doi: 10.2196/41839 (PMC9947755; doi:10.2196/41839)
Supplement: Multimedia Appendix 2 [file resprot_v12i1e41839_app2.pdf]

Information Sheet for Parent Volunteers

Research project name: Physiological and Psychological Effects of Parental Traditional Thai Massage on Children with Autism: a Randomized Controlled Trial

Research Project Leader:

Mr. HUI RUAN, Ph.D. Student — Exercise and sports science, graduate school, Khon Kaen university, Thailand

Co-researchers:

Assoc. Prof. Dr. Wichai Eungpinichpong, Ph.D. — School of Physical Therapy, Faculty of Associated Medical Sciences, Khon Kaen University, Thailand

Chanada Aonsri, Physical Therapy — Department of Special Education, Khon Kaen University Demonstration School, Faculty of Education, Khon Kaen University, Thailand

Funded from: Funded by the Research and Service Institute for Autism Development Program of Khon Kaen University in 2021 Funded by the Humanities and Social Sciences Foundation of the Ministry of Education of China in 2018

Introduction: Traditional Thai massage (TTM) can change symptoms in children with autism, but a previous study using professional massage therapists and a previous study using parental massage intervention was an exploratory study with no control group.

Objective of this study:

This randomized controlled trial will investigate the effect of parental TTM on the core symptoms of autism, as well as the influence of gait, heart rate variability, and parental stress in children with autism, and will explore their connections and possible mechanisms.

Voluntary participation in a research project or withdrawing from research:

You and your child are cordially invited to participate in our study. If you and your child can not participate in our study, your child's life and learning in school will not be affected in any way. If your child will participate in our study, they can always quit.

Details to be treated with program participants:

If you and your child decide to participate in the research and have signed the evidence for the volunteer consent form, forty-eight children with autism will be randomly divided into two groups. In addition to maintaining daily school routine, the intervention group would also receive will receive a total of 16 TTM interventions for 50 minutes twice a week for eight weeks. The control group simply maintained daily school

routine. After collecting the data, the parents of the control group will be given training in TTM. The details are as follows:

1. Parent volunteers

1) You will received traditional Thai massage for 3-hours/ session for 3 session in their children school by primary investigator or researcher.

2) In the first month of new semester, recruitment and other preparations will be completed, the experiment will begin in October. One or two days before the first intervention at the respective school, you (parent volunteer) will be asked to complete basic demographic information and the ATEC scale, which takes approximately 15 minutes, and will be asked to complete PSI-4-SF, which takes approximately 10 minutes.

3) Parents will perform massages on their children at around 3 p.m. in the health room of their school and researchers will be on hand to supervise. If your child will be random to the control group, your chill will receive the daily school activities.

4) After 16 interventions, the parent volunteer will be asked to complete the ATEC scale, which takes approximately 15 minutes, and will be asked to complete the PSI-4-SF, which takes approximately 10 minutes.

5) One month after the end of the entire experiment, during the fellow-up phase, the parent volunteers will be asked again to complete the ATEC scale, which take about 15 minutes, and will be asked to complete the PSI-4-SF scale, which takes about 10 minutes.

2. Teacher volunteers

Your children will be assess the ATEC by their teacher for 10 minutes for 3 time of assessment (before, after 16 interventions and 1 mount after)

3. Autistic children Volunteers

The intervention group will receive the first TTM intervention from your parents at school, you will be asked to test HRV and gait. HRV test takes about 5 minutes, And Gait test takes about 10 minutes, including putting on the device, corrosion and testing.

From sessions 2 to 15, the autistic children volunteers will receive TTM from the same room at their school in intervention group.

After 16 interventions, you will be asked to test HRV and gait. HRV test takes about 5 minutes, And Gait test takes about 10 minutes, including putting on the device, corrosion and testing.

For the children volunteers (control group), one or two days before the first intervention at the respective school, all autistic child volunteers will be asked to test HRV and gait. HRV test takes about 5 minutes, and gait test takes about 10 minutes, including putting on the device, adjusting and testing. After that, the control group will receive the first daily school activities, all autistic child volunteers will be asked to test HRV and gait. HRV test takes about 5 minutes, And Gait test takes about 10 minutes, including putting on the device, corrosion and testing. From sessions 2 to 15, will receive daily school activities

Potential risks and/or discomfort: The data collection did not cause any harm to you. But, you have to spend your time for training, evaluating and applying massage for your child.

Benefits that volunteers will receive:
By participating in this study, you will not receive any benefit from this study, but your children may receive the in-directed benefit from health assessment (HRV and gait) to know your current health status.

Research expenses/travel compensation/lost time allowance: This study will target you and your child volunteers in the intervention group, 50 yuan for each time, will be paid for each time to participate in the activity, a total of 16 times, another three training 100 yuan for each time, a total of 1100 yuan. In the control group, the training was 100 yuan for three times and the evaluation was 50 yuan for four times, a total of 500 yuan.

Confidentiality: The research group will keep your data confidential, and the research group will use the volunteers' passwords to record data, access data, etc. In this study, only the research group will know the data, and the research group will not state the names of the volunteers under any circumstances if the study results are published in the journal.

Whom to contact
If in doubt about the research details Please contact the research team:

Mr. HUI RUAN, PhD candidate, The Scientific Research Office of Hainan Normal University at 99 Longkun South Road, Haikou City. TEL: 18084699997

If you are treated incorrectly while participating in this study, or would like to know about your rights, please contact as following:

Center for Ethics in Human Research, Khon Kaen University, 17th floor, Sor Vor 1 building, 123 Mittraphap road, Nai Muang Subdistrict, Muang District, Khon Kaen, Thailand 40002
Tel. 043-366621-3 Mobile 089-7141177, 089-7141913 email: eckku@kku.ac.th

Consent form for parents

I (Mr., Mrs., Miss) First name..Last name..Age... ....year
Address..
Their children First name..Last name..Age................year
An explanation from ....About volunteering in the research project on Physiological and Psychological Effects of Parental Traditional Thai Massage on Children with Autism: a Randomized Controlled Trial detailed a research project on

- Objectives and duration of research
- Procedures and methods of conduct that I must follow
- The benefits that I will receive
- Side effects or dangers that may arise from participating in the program (specify as appropriate in accordance with the nature of the project) and I can withdraw from this study at any time if I wish. without forfeiting any right to receive medical care that will occur in the future, both now and in the future and if side effects occur, I will report to the researcher immediately.

I have read and understood the above explanation. Therefore, they have signed their consent to be a volunteer of the research project.

Volunteer's
signature ....
(...)
day/month/year ....

Informant's
signature ....
(...)
day/month/year ....

Principal researcher's
signature ....
(Hainan)
day/month/year ....

家长知情同意书

研究项目名称：父母传统泰式按摩对自闭症儿童生理和心理的影响:一项随机对照试验

研究负责人： 阮辉

研究对象：6 到 12 岁自闭症儿童

项目资助：2021 年由孔敬大学自闭症发展项目研究与服务研究所资助

2018 年中国教育部人文社会科学基金资助

引言： 传统泰式按摩(TTM)可以改变自闭症儿童的症状，但之前有一项使用专业按摩师的研究和一项使用家长按摩干预的研究，但是是没有对照组的探索性研究。

研究目的：本随机对照试验将探讨父母 TTM 对自闭症儿童核心症状的影响，以及步态、心率变异性和父母压力对自闭症儿童的影响，并探讨它们之间的联系和可能的机制。

您的研究项目参与者是自愿参加的：我们诚挚地邀请您和您的孩子参加我们的研究。如果您和您的孩子不能参加我们的学习，您的孩子在学校的生活和学习不会受到任何影响。如果孩子参与我们的研究，他也可以随时退出。

如果不参加项目则另行选择：如果您监护的孩子因任何原因不愿意继续研究，或者如果家长不愿意继续，研究将立即终止。

如果您参加研究项目,请采取行动：48 名自闭症儿童将被随机分为两组。除了维持学校的日常生活，干预组还将接受总共 16 项 TTM 干预，每次 50 分钟，每周两次，共 8 周。对照组只是维持学校的日常生活。数据收集后，控制组的家长将接受 TTM 培训。具体情况如下：

1. 家长志愿者和教师志愿者

在各自学校进行第一次干预前一两天，所有家长志愿者将被要求完成基本人口信息和 ATEC 量表(大约 15 分钟)，并被要求完成 PSI-4-SF 量表(大约 10 分钟)。教师志愿者将被要求完成 ATEC 量表，该量表大约需要 10 分钟乘以自闭症儿童的数量。然后在干预组，干预组的家长在学校，在通风良好，有衬垫的教室，室内温度为 25 度，对自闭症儿童进行第一次 TTM 干预。如果 ATEC 评估老师在场，他或她将被要求退出。

从第 2 次到第 15 次，在发明组，家长将在每个学校的同一个房间接受 TTM 干预，研究人员将在场监督。

在 16 次干预后，家长志愿者将被要求完成 ATEC 量表，大约需要 15 分钟，将被要求完成 PSI-4-SF 量表，大约需要 10 分钟。教师志愿者将被要求完成每个自闭症儿童的 ATEC 量表，所需时间将自闭症儿童的数量乘以每个 10 分钟。

在整个实验结束一个月后，在随访阶段，将再次要求家长志愿者完成 ATEC 量表，大约需要 15 分钟，将要求完成 PSI-4-SF 量表，大约需要 10 分钟。教师志愿者也将再次被要求完成每个自闭症儿童的 ATEC 量表，所需的时间将自闭症儿童的数量乘以每个 10 分钟。

2. 自闭症儿童的志愿者

在学校进行第一次干预前一两天，所有自闭症儿童志愿者将被要求测试 HRV 和步态。HRV 测试约 5 分钟，步态测试约 10 分钟，包括穿戴、调整和测试。

干预组将在学校接受父母的第一次 TTM 干预，所有自闭症儿童志愿者将被要求测试 HRV 和步态。HRV 测试大约需要 5 分钟，步态测试大约需要 10 分钟，包括穿戴设备和测试。

干预组的自闭症儿童志愿者将在他们学校的同一间教室接受 TTM 治疗。

在 16 次干预后，所有自闭症儿童志愿者将被要求测试 HRV 和步态。HRV 测试大约需要 5 分钟，步态测试大约需要 10 分钟，包括穿戴设备和测试。

潜在的风险和/或不适：本研究的数据收集对身体没有任何风险,因为 TTM 疗法是通过父母的志愿者,通过专家培训和适当的调整,使孩子放松和舒适此外,数据收集没有造成身体上的伤害儿童的暴力,没有心理、社会、金融风险。在进行实验之前，研究人员和家长会给孩子一些地方和时间放松，保持头脑清醒。在干预过程中，研究人员会安排一个良好的环境，一个舒适的温度，让孩子感到舒适、平静和私密。当数据收集时，父母将与研究人员在孩子身边，如果孩子没有准备好或不舒服，研究人员将在孩子准备好接受评估时重新评估。

志愿者能得到的好处: 通过参与这项研究，有可能改善自闭症儿童的核心症状，步态，也有可能改善自闭症儿童父母的压力和亲子关系。这可能证明泰式父母按摩是一种治疗自闭症的方法。

研究费用/旅行补偿费/耗费: 本研究将针对干预组中的儿童志愿者和家长志愿者，每次 50 元，将支付每次参加活动的费用，共 16 次，另外 3 次培训每次 100 元，共 1100 元。对照组培训 100 元 3 次，评价 50 元 4 次，共 500 元。

保守秘密: 研究小组将对儿童的数据保密，研究小组将使用志愿者的密码来记录数据、访问数据等。在本研究中，只有研究小组知道数据，如果研究结果发表在期刊上，研究小组在任何情况下都不会公布志愿者的姓名。如果您和您的孩子在参与本次研究过程中受到了不正确的对待，或

者您想了解您孩子的权利，请联系海口市龙昆南路 99 号海南师范大学科研室。

如阁下对研究工作有任何疑问,请联络:

海南师范大学 阮辉，电话：18084699997
